# Notoginsenoside R1 Reverses Abnormal Autophagy in Hippocampal Neurons of Mice With Sleep Deprivation Through Melatonin Receptor 1A

**DOI:** 10.3389/fphar.2021.719313

**Published:** 2021-09-16

**Authors:** Yin Cao, Qinglin Li, An Zhou, Zunji Ke, Shengqi Chen, Mingrui Li, Zipeng Gong, Zhengtao Wang, Xiaojun Wu

**Affiliations:** ^1^ Key Laboratory of Xin’an Medicine, Ministry of Education, Anhui Province Key Laboratory of R&D of Chinese Medicine, Anhui University of Chinese Medicine, Hefei, China; ^2^ Shanghai Key Laboratory of Compound Chinese Medicines, Institute of Chinese Materia Medica, Shanghai University of Traditional Chinese Medicine, Shanghai, China; ^3^ State Key Laboratory of Functions and Applications of Medicinal Plants, Guizhou Provincial Key Laboratory of Pharmaceutics, Guizhou Medical University, Guiyang, China; ^4^ Academy of Integrative Medicine, Shanghai University of Traditional Chinese Medicine, Shanghai, China

**Keywords:** sleep deprivation, autophagy, melatonin receptor 1A, learning and memory, notoginsenoside R1

## Abstract

Sleep deprivation (SD) may cause serious neural injury in the central nervous system, leading to impairment of learning and memory. Melatonin receptor 1A (MTNR1A) plays an important role in the sleep regulation upon activation by melatonin. The present study aimed to investigate if notoginsenoside R1 (NGR1), an active compound isolated from *Panax notoginseng*, could alleviate neural injury, thus improve impaired learning and memory of SD mice, as well as to explore its underlying action mechanism through modulating MTNR1A. Our results showed that NGR1 administration improved the impaired learning and memory of SD mice. NGR1 prevented the morphological damage and the accumulation of autophagosomes in the hippocampus of SD mice. At the molecular level, NGR1 reversed the expressions of proteins involved in autophagy and apoptosis, such as beclin-1, LC3B, p62, Bcl-2, Bax, and cleaved-caspase 3. Furthermore, the effect of NGR1 was found to be closely related with the MTNR1A-mediated PI3K/Akt/mTOR signaling pathway. On HT-22 cells induced by autophagy inducer rapamycin, NGR1 markedly attenuated excessive autophagy and apoptosis, and the alleviative effect was abolished by the MTNR1A inhibitor. Taken together, NGR1 was shown to alleviate the impaired learning and memory of SD mice, and its function might be exerted through reduction of excessive autophagy and apoptosis of hippocampal neurons by regulating the MTNR1A-mediated PI3K/Akt/mTOR signaling pathway.

## Introduction

Sleep is an essential and basic physiological activity for optimal body performance and health (Chen et al., 2020). Lack of sleep or sleep deprivation (SD) can lead to significant impairment of cardiovascular, immune, and endocrine health, as well as the central nervous system (Xie et al., 2013). Hippocampus plays a critical role in the modulation of memory, navigation, and cognition (Girardeau et al., 2017), and SD has been revealed to impair cognition, accompanied with ultrastructure damage and pyramidal neuron loss in the hippocampus (Xie et al., 2021).

Autophagy is a highly conserved cellular catabolism process that acts within cells to achieve substance equilibrium by recycling or degrading proteins and destroying organelles in lysosomal compartments (Lv et al., 2020). It is necessary for maintaining normal neuronal function and counteracting the abnormal changes of neurodegeneration, but dysfunctional autophagy destroys the homeostasis of neuronal cells and makes individuals prone to neurodegenerative or neuropsychiatric disorders (Hylin et al., 2018). When the autophagy process is regulated properly, it promotes cell survival, while dysregulated excessive autophagy leads to autophagic cell death (Yang et al., 2012). Our previous study found that abnormal autophagy activity in hippocampal neurons caused by SD is related to the impaired cognition; and when the excessive autophagy was suppressed through the PI3K/Akt/mTOR signaling pathway, the neuronal apoptosis in the hippocampus was relieved, implicating an important role of neuronal autophagy in SD-induced neurodegeneration (Cao et al., 2019; Cao et al., 2020).

Melatonin, known as the “dark hormone” in mammals, is mainly secreted by the pineal gland, exhibiting characteristic patterns of daily and seasonal secretion and playing an important role in sleep regulation (Tosches et al., 2014; Hu et al., 2016). It has potential therapeutic effect on neurodegenerative diseases (Pandi-Perumal et al., 2013). Recent studies have reported that melatonin can regulate autophagy through the PI3K/Akt/mTOR pathway (Pandi-Perumal et al., 2013). Melatonin reduction was found to be induced by SD (Pandi-Perumal et al., 2013), while compensatory melatonin can relieve the nerve injury under SD (Lan et al., 2001). Melatonin receptor 1A (MTNR1A) is one of the receptors for melatonin to exerts it neuroprotective function in sleep disorders (Lan et al., 2001).

Notoginsenoside R1 (NGR1) is a saponin extracted from P*anax notoginseng*, which possesses numerous beneficial properties, including antioxidant (He et al., 2012), anti-inflammatory (Son et al., 2009), cardioprotective (Ge et al., 2016), and neuroprotective (Meng et al., 2014) effects. It can increase neuronal excitability and ameliorate synaptic and memory dysfunction induced by amyloid elevation (Yan et al., 2014), and NGR1 exhibits neuroprotection by inhibiting neuronal apoptosis and promoting cell survival *via* PI3K-Akt-mTOR/JNK signaling pathways in neonatal cerebral hypoxic-ischemic brain injury (Tu et al., 2018). However, whether NGR1 can improve neuronal injury induced by SD has not been elucidated. Here, we explored the neuroprotective effects of NGR1 on mice induced by SD and tried to uncover its underlying molecular mechanism from the aspect of alleviating excessive autophagy and apoptosis through the MTNR1A-mediated signaling pathway.

## Materials and Methods

### Reagents

Notoginsenoside R1 (purity > 98%), rapamycin, and 3-methyladenine (3-MA) were bought from Dalian Meilun Biotechnology Co. Ltd. (Dalian, Liaoning, China). Selective MTR1A inhibitor was provided by Master of Small Molecules (MCE, Cat No: S26131). Antibodies against beclin-1 (A7353), LC3B (A19665), phosphoinositide 3-kinase (phospho-PI3K, AP0854), phospho-Akt (AP0098), PI3K p85 (A11402), Akt (A18120), Bax (A19684), cleaved caspase-3 (A19654), p62 (A11250), mTOR (A2445), MTNR1A (A13030), and β-actin (AC026) were purchased from ABclonal Biotechnology Co. Ltd. (Wuhan, Hubei, China). Bcl-2 antibody (abs131701) was bought from Apicent Biological Technology Co. Ltd. (Shanghai, China). p-mTOR antibody (5536S) was obtained from Cell Signaling Technology (Danvers, MA, United States).

### Animals and Treatment

Sixty male C57BL/6 mice (aged 6 weeks, weighing 18–22 g) were equally and randomly divided into six groups: control group, SD group, SD + modafinil (13 mg/kg) group, and SD + NGR1 groups (6.25 mg/kg, 12.5 mg/kg, and 25 mg/kg). Mice in each group, except the control group, were given respective drugs by gavage for 9 days after adaptive feeding for a week. Except for the control group, the mice in each group were sleep-deprived for 48 h on the eighth and ninth days according to a modified multiple platform method, as reported previously (Tu et al., 2018). All the animals were offered by Animal Research Center of Anhui University of Traditional Chinese Medicine and complied with a protocol approved by the Experimental Animal Ethics of Anhui University of Traditional Chinese Medicine with an approval number (AHUCM-mouse-2020041).

### Morris Water Maze Test

The learning and memory ability of mice was examined by the Morris water maze test. The procedure included adaptive training, visible training, hidden platform training, and space exploring, as reported previously (Suchecki and Tufik, 2000). Before space exploration, the mice, except those in the control group, were sleep-deprived for 48 h using a modified multiple platform method. After the behavioral test, the mice were euthanized with overdose of 1.5% pentobarbital sodium.

### Histopathological Analysis and Immunohistochemistry

Brain tissues of mice were dissected and fixed in 4% of paraformaldehyde solution for 24 h. For immunohistochemistry, the tissues were washed with PBS and soaked in 10 and 30% sucrose solution, respectively, for 24 h. Afterward, the tissues were embedded, frozen, and cut into 20-μm sections. After the infiltration and blocking for 30 min, the sections were incubated with a primary antibody against LC3B overnight at 4°C, followed by the incubation with a secondary antibody conjugated with Alexa Fluor 488. Fluorescent images were taken using the Olympus VS120 virtual slide scanner.

### Transmission Electron Microscopy

Following perfusion with phosphate-buffered saline (PBS) and fixation with 2% glutaraldehyde, the hippocampal tissues were cut into ultrathin sections. After dehydration, the sections were stained with uranyl acetate and lead citrate. The ultrastructure images were taken under an HT-7700 transmission electron microscope (Hitachi, Tokyo, Japan).

### Cell Culture and Treatment

Mouse HT-22 cell line was obtained from Cell Bank of Type Culture Collection of Chinese Academy of Sciences (Shanghai, China). The cells were maintained in DMEM basic medium, supplied with 10% fetal bovine serum and 1% penicillin/streptomycin. Prior to the drug treatment, the cells were seeded at a density of 5 × 10^5^ cells/ml in cell culture flasks or 24-well plates and cultured overnight. After pretreated with rapamycin (50 μg/ml) for 4 h, the cells were incubated with a complete medium: 3-MA (2.5 mM) or NGR1 at concentrations of 10, 20, and 40 μM for 48 h. Then the cells were lysed in lysis buffer with protease and phosphatase inhibitors for further Western blotting analysis.

### Acridine Orange Staining

HT-22 cells were cultured in 24-well plates and induced with rapamycin and treated with 3-MA or NGR1, as mentioned before. The medium was discarded, and the cells were rinsed with PBS. Afterward, the cells were incubated with acridine orange staining solution for 0.5 h at room temperature. The fluorescent images were taken under a fluorescence microscope. Generally, the normal cells were stained green uniformly, while the autophagic cells showed an orange cytoplasm.

### Flow Cytometry

HT-22 cells were induced with rapamycin and treated with 3-MA or NGR1, as described before. Then, the cells were collected with trypsin without EDTA. After washing twice with PBS, the cells were incubated with propidium iodide (PI, 2.5 μg/ml) and annexin V (2 μg/ml) for 10 min. Flow cytometry was carried out on a flow cytometer (Guava easy Cyte HT, Millipore, Germany).

### Western Blotting Analysis

Twenty microgram proteins from each sample were separated on 12% gel and transferred onto PVDF membranes. After blocking with nonfat milk solution (5%) for 1 h, the membranes were incubated with respective primary antibodies overnight at 4°C. Thereafter, they were washed with PBST and incubated with secondary antibodies for 1 h at room temperature. The protein bands were observed by an ECL Prime kit and quantified with ImageJ 1.46r software.

## Statistical Analysis

All the data were presented as mean ± standard error of the mean. One-way analysis of variance (ANOVA) with Dunnett post hoc analysis was performed to analyze the differences among the groups using GraphPad Prism 5.0. A *p* value < 0.05 was regarded as statistically significant.

## Results

### NGR1 Counteracted the Impairment of Learning and Memory of SD Mice

**FIGURE 1:**
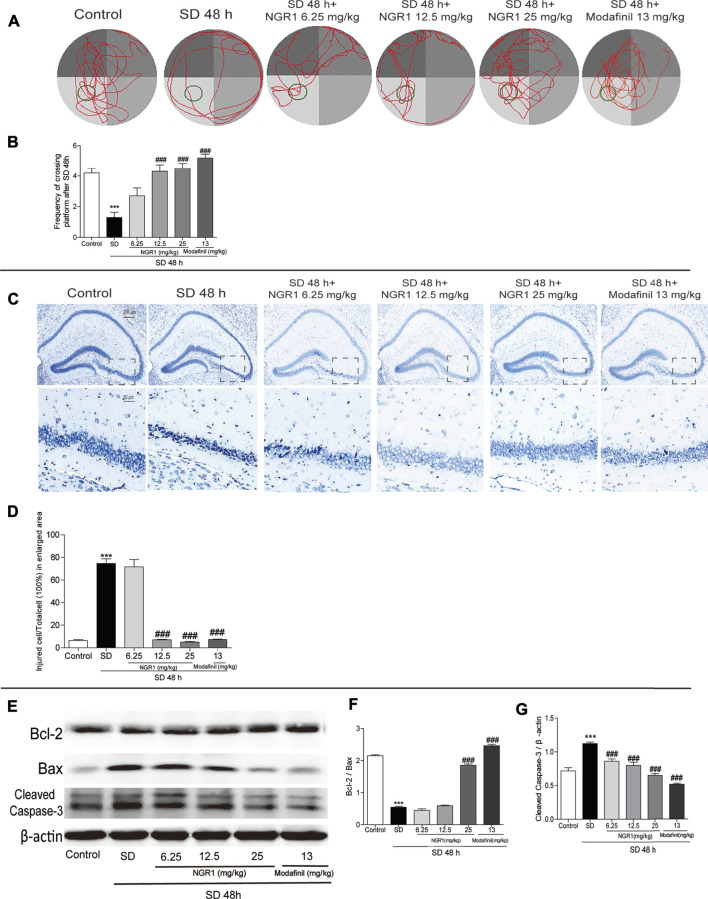
NGR1 counteracted the impairment of learning and memory of SD mice. **(A)** Movement tracks of mice in the Morris water maze. **(B)** Comparison of the frequency of mice passing through the target quadrant. **(C)** Nissl staining of hippocampal regions. The enlarged images displayed hippocampal CA3 regions. Scale bar: 200 and 50 µm **(D)** Comparison of the percentage of injured cells in CA3 regions. n = 3/group. **(E)** Hippocampal expressions of proteins involved in apoptosis. **(F–G)** Gray intensity comparison of Bcl-2/Bax and cleaved caspase-3. n = 5/group. ***p < 0.001 vs. control group. ###*p* < 0.001 vs. SD group. (NGR1: notoginsenoside R1; SD: sleep deprivation).

As shown in Figures 1A,B, mice sleep-deprived for 48 h visited the target quadrant (hidden platform) less than the control ones (*p* < 0.001). By contrast, after NGR1 treatment, especially at 12.5 mg/kg and 25 mg/kg, the SD mice displayed significantly increased frequency to explore the target quadrant compared with the model group mice (*p* < 0.001). Mice in the modafinil-treated group also showed significantly improved learning and memory capacity (*p* < 0.001). Accordingly, the morphology of the pyramidal cells in CA3 region of the hippocampus of SD group mice changed dramatically. As shown in Figure 1C, the cells were shrunken to an irregular shape and stained in deep blue color. NGR1 and modafinil pretreatment significantly prevented the morphological changes of the pyramidal cells induced by SD (Figure 1D, *p* < 0.001). Moreover, SD caused the decrease in Bcl-2 but increase in Bax and cleaved caspase-3 in the hippocampus of mice (Figures 1E–G). NGR1, particularly at 25 mg/kg, and modafinil counteracted the effect of SD on the ratio of Bcl2/Bax and the expression of cleaved caspase-3 (*p* < 0.001). These results demonstrated that NGR1 pretreatment could alleviate the impairment of learning and memory of SD mice by reducing the neuronal injury.

### NGR1 Decreased the Excessive Neuronal Autophagy in the Hippocampus of SD Mice

**FIGURE 2:**
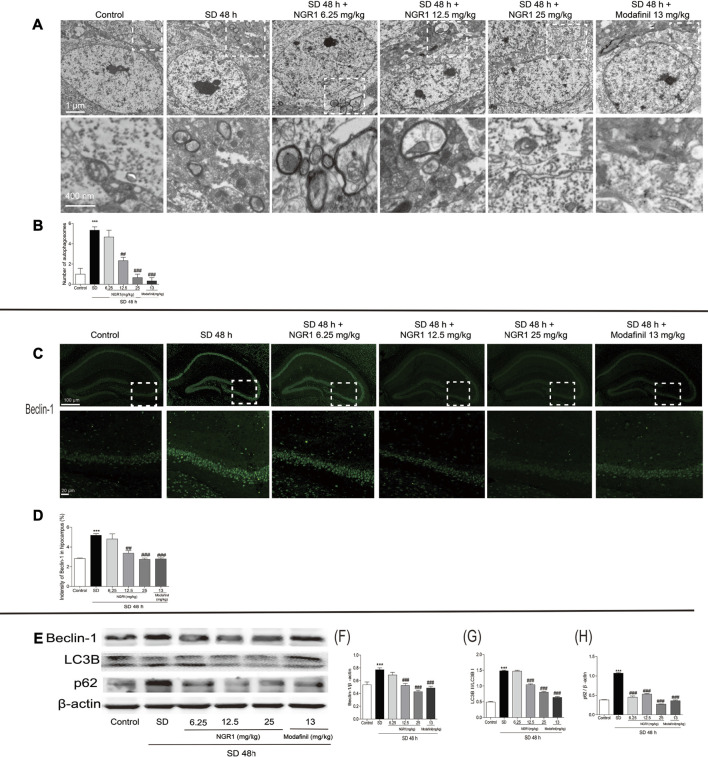
NGR1 suppressed the excessive autophagy in the hippocampus of SD mice. **(A)** Autophagosomes in hippocampi examined by transmission electron microscopy. The autophagosomes were denoted by arrows. Scale bar: 1 µm and 400 nm. **(B)** Comparison of the number of autophagosomes. n = 3/group. **(C)** Immunostaining of beclin-1 in the hippocampus. Scale bar: 100 and 20 µm. **(D)** Comparison of beclin-1 immunofluorescence intensity. n = 3/group. **(E)** Hippocampal expressions of proteins involved in autophagy. **(F–H)** Gray intensity comparison of beclin-1, LC3BII/I, and p62. n = 5/group. (NGR1: notoginsenoside R1; SD: sleep deprivation; p62, and ubiquitin-binding protein p62 or sequestosome-1).

As shown in Figure 2A, in hippocampal CA3 region of SD mice, the number of autophagosomes increased significantly (*p* < 0.001) compared with the control group. When pretreated with NGR1 (12.5 mg/kg and 25 mg/kg) and modafinil, the numbers were reduced markedly (*p* < 0.01 or *p* < 0.001). In agreement with the changes of autophagosomes, after NGR1 pretreatment, the immunofluorescence intensity of beclin-1 decreased, especially at 12.5 25 mg/kg and 25 mg/kg groups (Figures 2C,D, *p* < 0.01 or *p* < 0.001). Furthermore, the protein expressions of beclin-1 and p62 as well as the ratio of LC3BII to LC3BI were all increased in the hippocampus of SD mice, which could be reversed by NGR1 and modafinil pretreatment (Figures 2E–H). These results demonstrated that NGR1 could reduce autophagy of hippocampal neurons in SD mice.

### NGR1 Increased the Expression of MTNR1A and Enhanced Its Downstream Signaling in the Hippocampus of SD Mice

**FIGURE 3:**
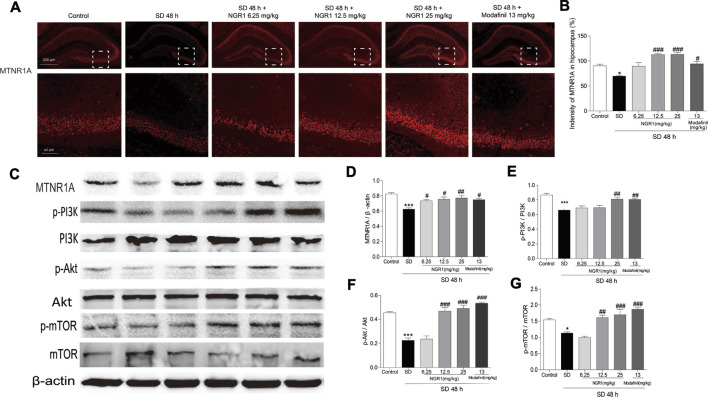
NGR1 resumed the decreased MTNR1A in the hippocampus induced by SD. **(A)** Immunostaining of MTNR1A in the hippocampus. Scale bar: 200 and 50 µm. **(B)** Comparison of immunofluorescence intensity of MTNR1A in the hippocampus. n = 3/group. **(C)** Protein expression of MTNR1A in hippocampi. n = 5/group. **(D–G)** Protein expressions of the phosphorylated PI3K, Akt, and mTOR in hippocampi. n = 5/group. *p < 0.05; ***p < 0.001 vs. control group. ##*p* < 0.01, ###*p* < 0.001 vs. SD group. (NGR1: notoginsenoside R1; MTNR1A: melatonin receptors 1A; SD: sleep deprivation; mTOR, mammalian target of rapamycin; p-Akt, phosphorylated PKB; p-PI3K, phosphorylated PI3K).

As shown in Figures 3A,B, the immunofluorescence intensity of MTNR1A was reduced significantly in the hippocampus of SD mice (*p* < 0.05). However, after NGR1 pretreatment (12.5 mg/kg and 25 mg/kg), the immunofluorescence intensity of MTNR1A was increased (*p* < 0.001). Consistently, as revealed in Figure 3C, the protein expression of MTNR1A was mitigated in the hippocampus of SD mice. Meanwhile, the phosphorylation of the downstream signaling molecules, such as PI3K, AKT, and mTOR, was also reduced significantly, compared with the control mice (*p* <0.05 or *p* <0.001). After NGR1 pretreatment, the changes in the expression of MTNR1A and its downstream signaling pathway molecules induced by SD were abrogated. These results indicated that NGR1 might exert its function through the MTNR1A-mediated signaling pathway.

### NGR1 Reduced the Excessive Autophagy and Apoptosis Induced by Rapamycin in HT-22 Cells

**FIGURE 4:**
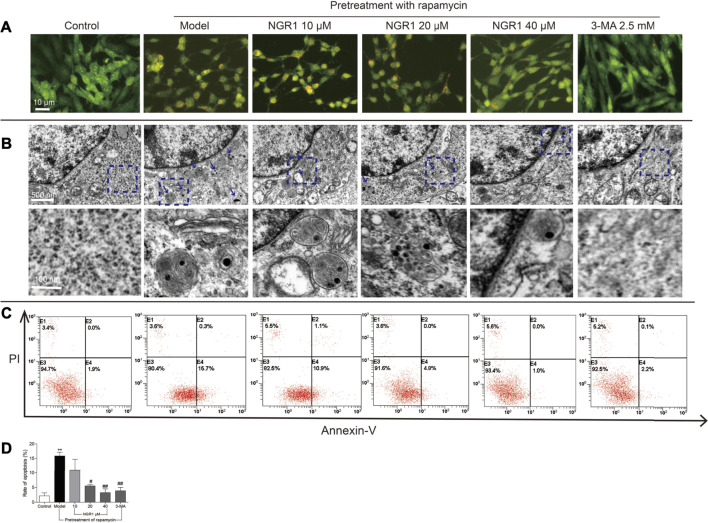
NGR1 reduced autophagy and apoptosis of HT-22 cells induced by rapamycin. **(A)** Acidic vesicular organelles stained by acridine orange. Scale bar: 10 µm **(B)** Autophagosomes in cells examined by transmission electron microscopy. The autophagosomes were denoted by arrows. Scale bar: 500 and 100 nm. **(C)** and **(D)** Apoptosis examined by flow cytometry after annexin V/PI staining. n = 3/group. **p < 0.01 vs. control group. #*p* < 0.05, ##*p* < 0.01 vs. model group. (NGR1: notoginsenoside R1; 3-MA, 3-methyladenine).

As shown in Figure 4A, rapamycin induced the accumulation of acidic vesicular organelles (AVOs) in orange color in HT-22 cells. NGR1, when used at higher concentrations (20 and 40 μM), significantly reduced the intensity of AVOs within the cells. 3-MA, an inhibitor of autophagy, showed similar effect as NGR1. Furthermore, there were more autophagosomes in rapamycin-induced model group cells. However, both NGR1 and 3-MA treatment could reduce the number of autophagosomes. In addition, as shown in Figures 4C,D, rapamycin increased the ratio of apoptotic cells (*p* < 0.01), which could be suppressed by the treatment of NGR1 (20 and 40 μM) and 3-MA (*p* < 0.05 or *p* <0.01). These results demonstrated that NGR1 could prevent excessive autophagy and apoptosis induced by rapamycin in HT-22 cells.

### NGR1 Reversed the Excessive Autophagy and Apoptosis Induced by Rapamycin in HT-22 Cells Through Regulating MTNR1A

**FIGURE 5:**
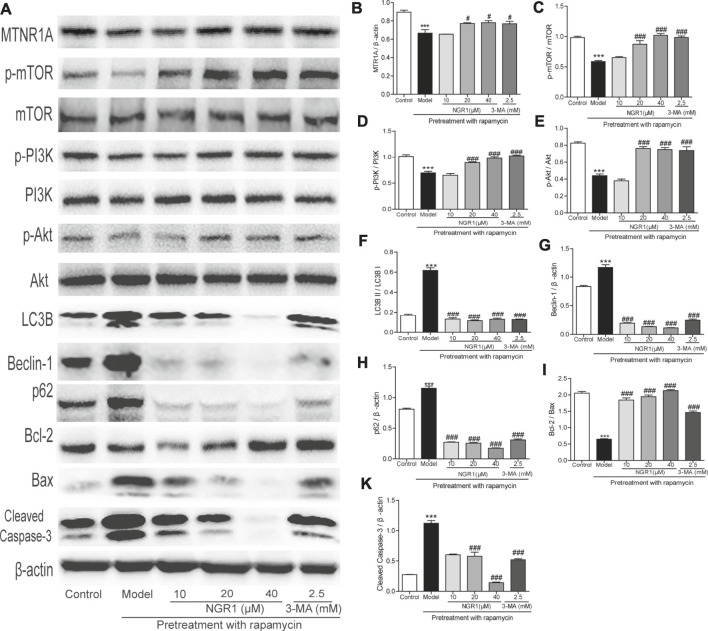
NGR1 regulated the expressions of proteins involved in autophagy and apoptosis in HT-22 cells induced by rapamycin. **(A)** Protein bands. **(B)**–**(K)** Gray intensity analysis of the protein bands. n = 5/group. ***p < 0.001 vs. control group. #*p* < 0.05, ###*p* < 0.001 vs. model group. (NGR1: notoginsenoside R1; MTNR1A: melatonin receptors 1A; 3-MA, 3-methyladenine; mTOR, mammalian target of rapamycin; p62, ubiquitin-binding protein p62 or sequestosome-1; p-Akt, phosphorylated PKB; p-PI3K, phosphorylated PI3K).

As shown in Figure 5, rapamycin increased the protein expression of beclin-1 and p62 as well as the ratio of LC3BII to LC3BI in HT-22 cells (*p* < 0.001). Meanwhile, it also decreased the ratio of Bcl-2/Bax but elevated the expression of cleaved caspase-3 (*p* < 0.001). Both NGR1 and 3-MA treatment could reverse the changes of the proteins involved in autophagy and apoptosis (*p* < 0.001). Moreover, NGR1 increased the expression of MTNR1A (*p* < 0.05) and enhanced the phosphorylation of PI3K, AKT, and mTOR in HT-22 cells induced by rapamycin (*p* < 0.001).

**FIGURE 6:**
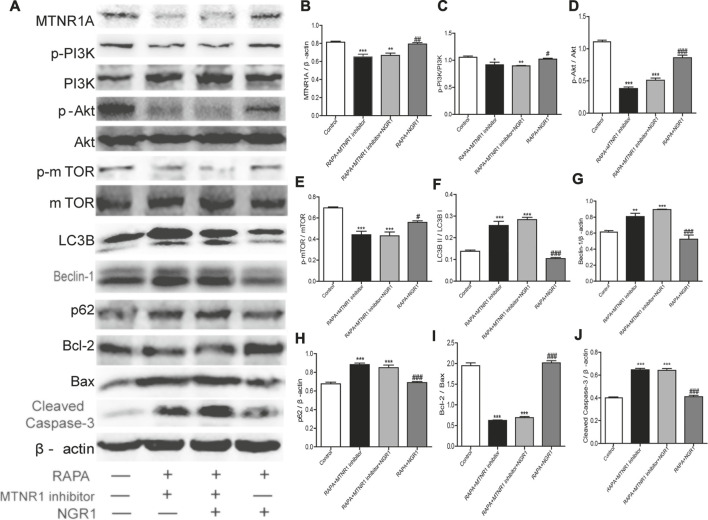
MTNR1A inhibitor abolished the alleviative effects of NGR1 on the expressions of proteins involved in autophagy and apoptosis in HT-22 cells induced by rapamycin. After being pretreated with rapamycin (50 µg/ml) for 4 h, the cells were exposed to NGR1 (20 μM) or NGR1 (20 μM) and the MTNR1 inhibitor (10 µM) for 48 h. **(A)** Protein bands. **(B)**–**(J)** Gray intensity analysis of the protein bands. n = ?/group. *p < 0.05, **p < 0.01, ***p < 0.001 vs. control group. #*p* < 0.05, ##*p* < 0.01, ###*p* < 0.001 vs. RAPA + MTNR1 inhibitor + NGR1 group. (NGR1: notoginsenoside R1; MTNR1: melatonin receptors 1; 3-MA, 3-methyladenine; mTOR, mammalian target of rapamycin; p62, ubiquitin-binding protein p62 or sequestosome-1; p-Akt, phosphorylated PKB; p-PI3K, phosphorylated PI3K).

To confirm the role of MTNR1A in the protective effect of NGR1, the MTNR1A inhibitor was used. As shown in Figure 6, when the inhibitor was added, the alleviative effect of NGR1 on the excessive autophagy and apoptosis induced by rapamycin was abolished in HT-22 cells.

## Discussion and Conclusion

SD can cause deficiency of learning and memory as it induces excessive autophagy and apoptosis in hippocampal neurons according to our previous studies (Suchecki and Tufik, 2000). In the present study, NGR1 was shown to attenuate the impaired learning and memory of SD mice. Moreover, it prevented the morphological changes of the pyramidal layer neurons and reduced excessive autophagy and apoptosis, evidenced by decreased autophagosomes and recovered abnormally expressed proteins, such as beclin-1, p62, LC3B, Bcl-2, Bax, and cleaved caspase-3, that involved in the processes of autophagy and apoptosis. These results clearly demonstrated that NGR1 could alleviate SD-induced hippocampal neuronal injury and thus improve the impaired learning and memory in mice.

Phosphoinositide 3-kinase (PI3K), an intracellular phosphatidylinositol kinase, consists of a catalytic subunit (p110) and a regulatory subunit (p85) (Hennessy et al., 2005), activates protein kinase B (Akt) and mammalian target of rapamycin (mTOR), and actively participates in the regulation of autophagy (Cantley, 2002) (Hennessy et al., 2005). Akt is a serine/threonine kinase and is a primary downstream target in the transduction pathway of PI3K signaling. It is a key signal molecule that promotes cell survival, inhibits apoptosis (Ouyang et al., 1999), and maintains normal functions (Castaneda et al., 2010). mTOR is a serine/threonine protein kinase that controls Atg genes and regulates autophagy negatively (Pattingre et al., 2008). Melatonin is reported to regulate autophagy through the PI3K/Akt/mTOR pathway (Roohbakhsh et al., 2018). Melatonin receptors, MTNR1A and MTNR1B, belong to GPCR family–coupled G proteins. Reducing the level of cAMP, the second messenger in cells, is the most commonly triggered signaling pathway by melatonin (Cecon et al., 2018). The neuroprotective effect of melatonin has been shown to be mediated by the melatonin receptor MTNR1A (Wang et al., 2011). A decrease in the MTNR1A level in the individuals has been revealed to increase the risk to aggravate the pathological process of Alzheimer’s disease (Sulkava et al., 2018).

In the present study, SD was shown to suppress the expression of MTNR1A in hippocampal tissue and inhibit the activation of the PI3K/Akt/mTOR pathway. After administration of NGR1, the suppression was alleviated. On HT-22 cells pretreated with rapamycin, NGR1 was demonstrated to reverse the reduced MTNR1A expression and inhibited the PI3K/Akt/mTOR pathway. To confirm the important role of MTNR1A, the inhibitor of MTNR1A was employed, which, at least partly, abolished the effect of NGR1. These results suggested that NGR1 exerted its neuroprotective role through acting on MTNR1A.

**FIGURE 7:**
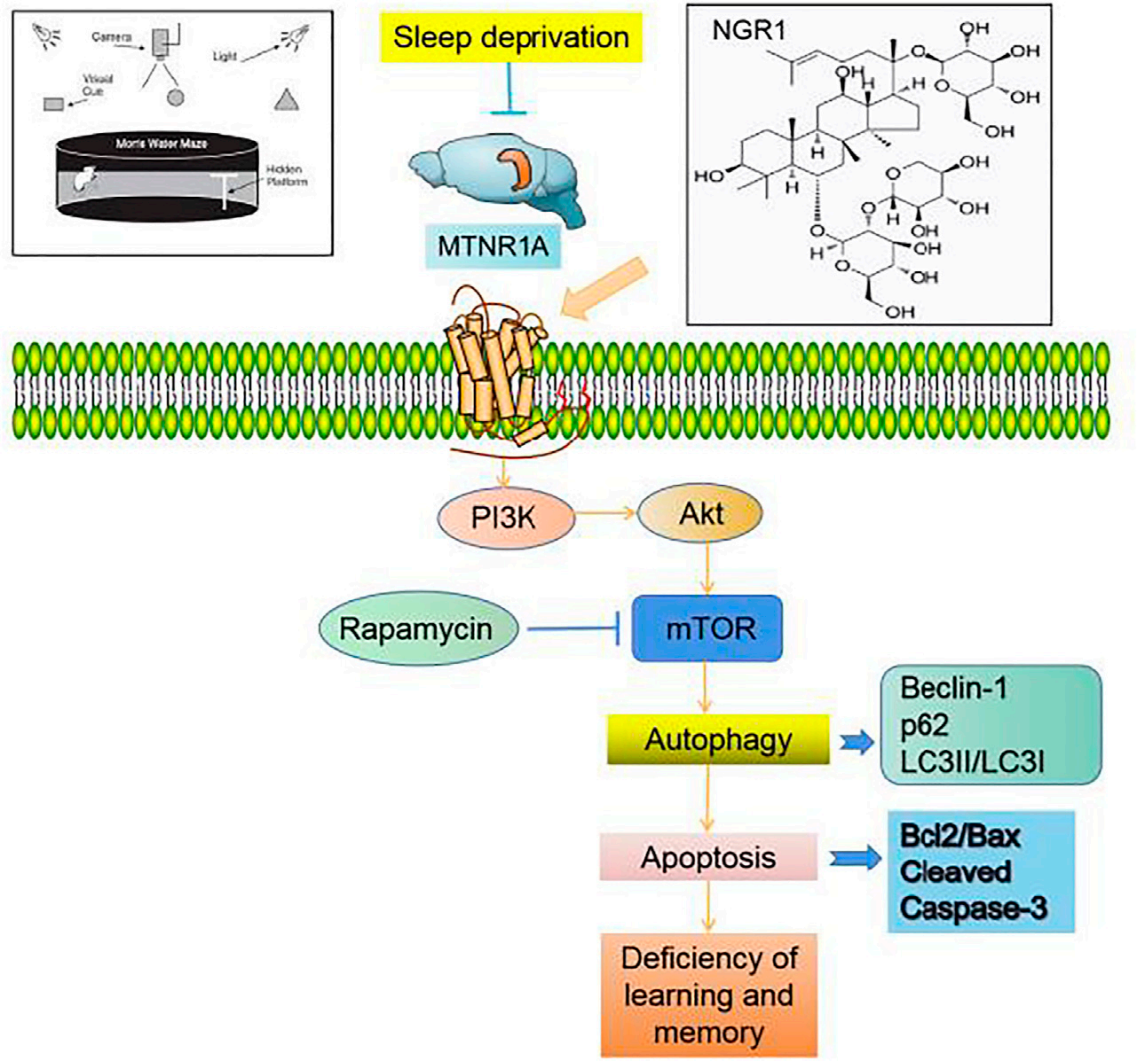
Notoginsenoside R1 reverses abnormal autophagy in hippocampal neuron of mice with sleep deprivation through melatonin receptor 1A.

In conclusion (Figure 7), NGR1 could attenuate the impaired learning and memory of SD mice, which might be exerted by inhibiting the excessive autophagy and apoptosis through MTNR1A-mediated PI3K/Akt/mTOR pathway in hippocampal neurons.

## Data Availability

The original contributions presented in the study are included in the article/Supplementary Material; further inquiries can be directed to the corresponding authors.
