# Corrigendum: Notoginsenoside R1 Reverses Abnormal Autophagy in Hippocampal Neurons of Mice With Sleep Deprivation Through Melatonin Receptor 1A

**DOI:** 10.3389/fphar.2021.832126

**Published:** 2022-01-19

**Authors:** Yin Cao, Qinglin Li, An Zhou, Zunji Ke, Shengqi Chen, Mingrui Li, Zipeng Gong, Zhengtao Wang, Xiaojun Wu

**Affiliations:** ^1^ Key Laboratory of Xin’an Medicine, Ministry of Education, Anhui Province Key Laboratory of R&D of Chinese Medicine, Anhui University of Chinese Medicine, Hefei, China; ^2^ Shanghai Key Laboratory of Compound Chinese Medicines, Institute of Chinese Materia Medica, Shanghai University of Traditional Chinese Medicine, Shanghai, China; ^3^ State Key Laboratory of Functions and Applications of Medicinal Plants, Guizhou Provincial Key Laboratory of Pharmaceutics, Guizhou Medical University, Guiyang, China; ^4^ Academy of Integrative Medicine, Shanghai University of Traditional Chinese Medicine, Shanghai, China

**Keywords:** notoginsenoside R1, sleep deprivation, autophagy, melatonin receptor 1A, learning and memory

In the original article, there was an error in the **Funding** statement. The correct number for the funder “Youth Project of Anhui Natural Science Foundation” is 2108085QH372, not 2108085QH3720. There was also a mistake in Figure 7, the chemical form of *NGR1* is incorrect. The correct Figure 7 appears below.

**FIGURE 7:**
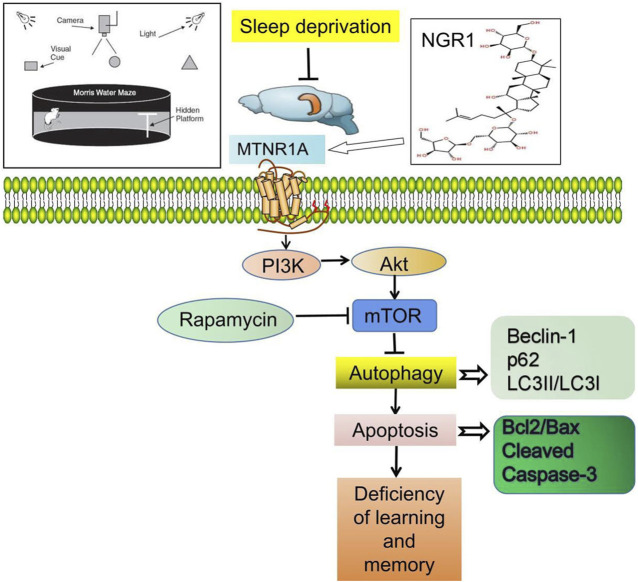
Notoginsenoside R1 reverses abnormal autophagy in hippocampal neuron of mice with sleep deprivation through melatonin receptor 1A.

The authors apologize for these errors and state that this do not change the scientific conclusions of the article in any way. The original article has been updated.

